# Sensitivity and Specificity of Hypnosis Effects on Gastric Myoelectrical Activity

**DOI:** 10.1371/journal.pone.0083486

**Published:** 2013-12-16

**Authors:** Paul Enck, Jochen Hefner, Beate M. Herbert, Nazar Mazurak, Katja Weimer, Eric R. Muth, Stephan Zipfel, Ute Martens

**Affiliations:** 1 Department of Psychosomatic Medicine, University Hospital, Tübingen, Germany; 2 Department of Internal Medicine II, University Hospital, Würzburg, Germany; 3 Department of Health Psychology, Institute of Psychology and Education, University of Ulm, Ulm, Germany; 4 Central Research Department, Ivano-Frankivsk National Medical University, Ivano-Frankivsk, Ukraine; 5 Department of Psychology, Clemson University, Clemson, South Carolina, United States of America; The University of Queensland, Australia

## Abstract

**Objectives:**

The effects of hypnosis on physiological (gastrointestinal) functions are incompletely understood, and it is unknown whether they are hypnosis-specific and gut-specific, or simply unspecific effects of relaxation.

**Design:**

Sixty-two healthy female volunteers were randomly assigned to either a single session of hypnotic suggestion of ingesting an appetizing meal and an unappetizing meal, or to relax and concentrate on having an appetizing or unappetizing meal, while the electrogastrogram (EGG) was recorded. At the end of the session, participants drank water until they felt full, in order to detect EGG-signal changes after ingestion of a true gastric load. During both conditions participants reported their subjective well-being, hunger and disgust at several time points.

**Results:**

Imagining eating food induced subjective feelings of hunger and disgust as well as changes in the EGG similar to, but more pronounced than those seen with a real gastric water load during both hypnosis and relaxation conditions. These effects were more pronounced when imagining an appetizing meal than with an unappetizing meal. There was no significant difference between the hypnosis and relaxation conditions.

**Conclusion:**

Imagination with and without hypnosis exhibits similar changes in subjective and objective measures in response to imagining an appetizing and an unappetizing food, indicating high sensitivity but low specificity.

## Introduction

Hypnosis is a tool used to induce deep relaxation that has been around since Charcot [1]. Hypnosis has maintained a questionable reputation not only because hypnotic quackery has determined its public appearance and opinion [2], but also since many different schools and traditions have confused professionals [3]. However, over the last 30 years, hypnotherapy has become a serious therapy in many medical, especially in psychiatric and psychotherapeutic areas, partially because of well-controlled clinical trials showing its efficacy [4-6].

In 1984, Whorwell and others [7] presented a clinical study that showed high efficacy of hypnotherapy over supportive psychotherapy and placebo therapy in patients with functional gastrointestinal disorders such as the irritable bowel syndrome (IBS). They used a novel hypnosis technique they called "gut-directed hypnosis" (GDH), that used pictures specific to the gastrointestinal tract following induction of hypnotic relaxation [8]. GDH has since shown remarkable short-term as well as long-tem clinical efficacy in many IBS studies [9,10], in adults as well as in children [11], and with individual therapy as well as in group settings [12].

Most studies have assessed only if hypnosis produces clinical responses (changes in symptom reports). Only a few studies have also examined the psychological and physiological responses to hypnosis that could explain its clinical effectiveness [13-16]. These studies explored changes in gastrointestinal motility and sensory functions measured prior to and after a series of hypnotherapy sessions. Physiological measurements during hypnosis – either gut-specific or unspecific – are thus far lacking.

Only a few bio-signal recording techniques allow direct measurement of intestinal functions without interference with the induction of hypnotic relaxation, i.e. continuous non-invasive data recording. Among them, the electrogastrogram (EGG) [17] provides a tool that allows the influence of sympathetic and parasympathetic activity on gastric activity to be observed [18,19]. To date, a few studies have examined the effects of imagining - favorable or unfavorable - food on the EGG [20-22]. These studies employed a paradigm that asked participants to imagine their most liked or disliked food.

The present study assessed whether hypnotic suggestion of gut-relevant images would induce acute and specific gastric responses as measured by the EGG, and if these responses would mimic responses that are known to occur with eating. To control for the hypnosis-specificity of responses, we compared hypnosis to an unspecific concentration task without induction of a hypnotic state. To explore the effects of the nature of the imagined food, we compared pleasant and unpleasant food imagination in a within-subject crossover design in both study arms. We also determined the EGG response to drinking water "to full" at the end of each session [23]. Because predominantly women are affected by the IBS condition [24], and in the GDH clinical studies by Whorwell and others predominantly women were included [10], female volunteers only were recruited for this pilot experiment. As previous data were not available to estimate an expected effect size, the hypnosis-arm of the study was enhanced as compared to the control arm at a ratio of 2:1 participants. 

Based on previously published papers [20-22] it was hypothesized that a more pronounced EGG response would be observed during hypnosis than with the concentration paradigm, and compared to the drinking test following hypnosis. We also hypothesized that imagination of an appetizing meal would have a distinctly different impact on subjective reports and the EGG compared to just focusing on an unappetizing meal, under both imagination conditions. Specifically, we expected the appetizing food to increase 3 cycles-per-minute (cpm) EGG activity at the expense of activity in the tachygastric band, and the unappetizing meal to increase tachygastria at the expense of 3 cpm activity.

## Method

The study was conducted during the summer of 2010. The study protocol was approved by the ethics board of the Medical Faculty Tübingen, and all volunteers gave written informed consent prior to participation.

### Participants

Sixty-three female volunteers were recruited via advertisements placed in medical school buildings; all were either students or medical center personnel. Exclusion criteria were any psychiatric or organic diseases reported during a medical interview and prescription medicine use other than contraceptives. One volunteer was familiar with the techniques of clinical hypnosis and was excluded from the analysis.

Prior to enrollment, volunteers were tested for hypnotic susceptibility using the Harvard Group Scale of Hypnotic Susceptibility [25]. This questionnaire based test was administered in groups of between 6 and 15 individuals on a separate occasion prior to the experiment. All participants were asked to fill out the German translation of Form A [25]. At this time, the participants also filled out the 34-item Tellegen Absorption Scale (TAS-2) [26] that measures the ability to concentrate (being "absorbed"), which has been proposed to be associated with hypnotic suggestibility [27].

**Figure 1:**
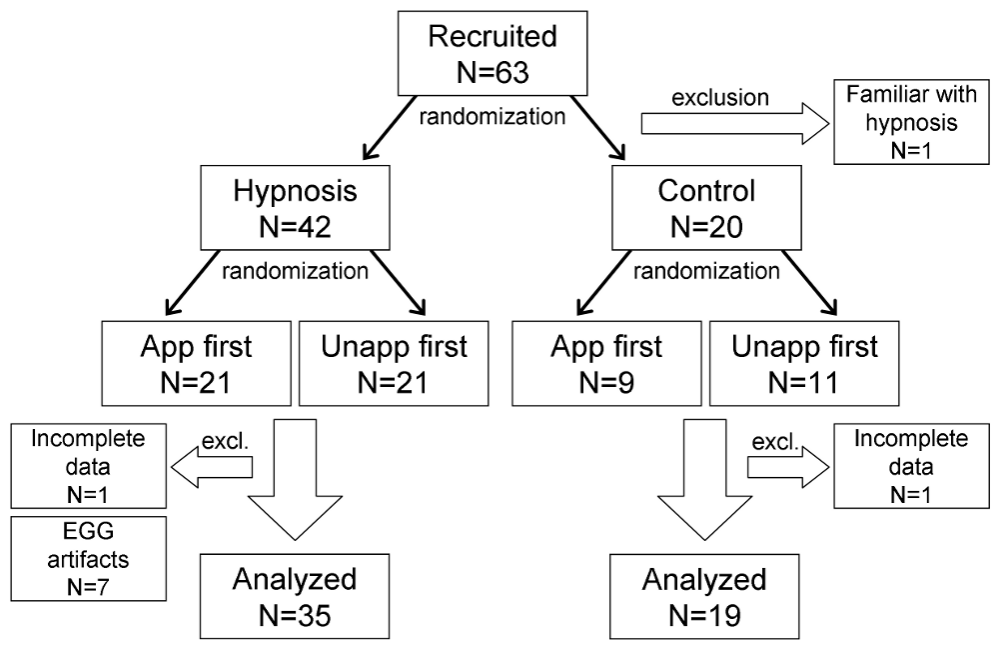
Recruitment and randomization scheme for the hypnosis study, with randomized sequence of imagination of either the appetizing meal (app) or the unappetizing meal (unapp) in both study arms.

Because of incomplete data, two participants were subsequently excluded from the analysis. Figure 1 shows a flowchart of recruitment and exclusion of volunteers.

### The experiment

Forty-two participants were randomly assigned to the hypnosis condition, and 20 participants to the concentration task. Participants were not made aware of their assignment. To enrich the number of volunteers in the hypnosis group, the randomization ratio was set to 2:1 for hypnosis and control, respectively.

On their respective test day, volunteers were asked to avoid caffeine and nicotine and fast for at least 3 hours before participating. Drinking water ad libitum was permitted. 

All measures were taken between 8.00h and 13.00h, and all participants were investigated by the same researchers (JH, NM). After preparation for EGG recording (see below), participants sat comfortably in a lounge chair, in a sound-attenuated and climate-controlled room. The participants were then introduced to the history and rationale of hypnotherapy without an explicit description of techniques used for its induction. 

### Hypnosis

A 10 minute baseline was collected prior to hypnotic induction by having participants sit and relax, with eyes closed. Following the baseline, the 10 minute hypnotic induction was performed by an experienced therapist (JH) using the following standard procedure [28].

*Hypnotic induction comprised inviting the participant to close their eyes and focus on perceptions of their body, neglecting distractions from their environment. The participant was invited to become aware of sensations from their lower extremities upwards, intensifying their current perceptions of their bodies. Following successive focus on the hips, torso, neck and head, participants were invited to focus on their hands. After a few moments of building up tension in both hands, participants were asked to relax and focus all of their sensations on their hands, and told that it signaled relaxation that could then spread throughout their body. After that, instructions of feeling increasingly relaxed while lying on, or sinking into a cloud were given. Finally, it was suggested that the unconscious mind would take care of all bodily functions so that participants could deeply relax and follow the suggestions.*

After hypnotic induction, a first assessment of the participant’s subjective well-being was performed. This was followed by listening to a 10-minute mp3-file recording of the same therapist (JH) that contained either suggestions of one’s favorite food (seeing the food, hearing it being prepared, smelling, tasting and eventually eating the food), or the participant’s most unappetizing (disgusting) food. This imagining period was followed by a second assessment of subjective well-being. This procedure was repeated a second time, for the opposite food imagined the first time (appetizing or unappetizing). After the final assessment of subjective well-being, hypnosis was terminated. Participants were then asked to drink clear water "to full“, i.e. to drink as much as they wanted within 5 minutes until they had a feeling of fullness [23].

Audio-taped instructions were used to standardize the procedure across all volunteers. This type of procedure has been validated against interpersonal instruction bias in previous research (e.g. 29,30). Most studies on IBS therapy use audiotapes to allow the patients to practice at home [31]. Others have proposed a stand-alone audio-taped home-treatment approach [28]. Audio-taped hypnosis is known to be effective, but less effective than the presence of a live hypnotherapist [32].

It has also been shown that 10 minutes are sufficient to induct a trance-like state and to provide vivid suggestions of a pleasant or unpleasant meal. Other studies have used shorter induction periods. One study examining the guided imagery of a threatening or non-threatening animal used an 8 minute induction period [33]. A study on emotional and autonomic reactions used 3 minute induction periods. In another study [34] on heart repolarization, subjects not tested for hypnotic susceptibility received suggestions of emotions and sensations (fear, anger, happiness, pain, humor) for 5 minute periods after 10 minutes of hypnotic induction. 

Imagery conditions were counter-balanced such that half of the volunteers received the imagining sequence appetizing-unappetizing, and for the other half it was reversed. Order assignment was randomized and balanced between conditions. Suggestions of the sensory qualities of the imagined food were presented without proposing a specific food or meal. 

### Control (concentration) task

For the control condition, the time course of events during relaxation was identical to the time course during hypnotic induction, with the procedure being that volunteers were simply asked to relax further every two minutes, i.e. the relaxation phase, equivalent in length to the 10 min hypnosis induction period. The same appetizing and unappetizing imagery and drink to full procedures were then followed as in the hypnosis condition.

**Figure 2:**
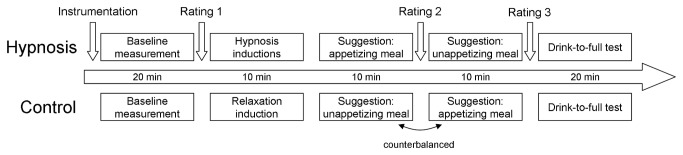
Time scale of the hypnosis study. All measurements were conducted between 8.00h and 13.00h in the morning.

Figure 2 shows the time scale of the experiment.

### Assessment of well-being

At predefined time-points (Q1 to Q4, see Figure 2), participants were asked to rate their current feelings on a 10-point Visual Analog Scale (VAS) (0 = not all, 10 = completely) with respect to the following seven items: pleasant, unpleasant, aroused, anxious, relaxed, disgusted and sick. The five negative items (unpleasant, anxious, relaxed, disgusted and sick) were reverse scored. Then all items were totaled to compute a well-being score ranging between 0 and 70. The items of hunger and satiety ("full") (similarly rated between 0 and 10) were separately used to evaluate the specific effects of the appetizing and unappetizing meals.

### The electrogastrogram (EGG)

Gastric myoelectrical activity was recorded by an electrogastrogram (EGG) for which three skin electrodes were placed above the stomach as described in the literature [37] and connected to a Biolog device with Fetrode technology (UFI, Morrow Bay, CA, USA). EGG data were collected at a sampling rate of 10 Hz. EGG data were analyzed using a Fast-Fourier running spectral analysis (using custom software based on Prime Factor FFT for Windows, version 3.03, Alligator Technologies, Costa Mesa, CA, USA). A Hamming window was applied to 2048 points of data and successive windows were overlapped by 75%. Spectral estimates from the multiple windows were averaged separately for each subject and each condition to result in a single series of estimates. Total power was calculated as a sum of the spectral estimates from 1-10 cycles per minute (cpm). Percentage of total power was calculated for the bradygastria (1,2), normogastria or 3 cpm, and tachyarrhythmia (4-9) bands (for detailed information see 17). The ratio between the percentage of the normogastria and the tachygastria band serves as an indicator for nausea. Ratio values above 1 indicate normal gastric activity and values below 1 indicate increased tachygastria. The interruption of the normal 3 cpm activity of the stomach and a shift towards tachygastria has been repeatedly associated with nausea, e.g. induced by a rotating chair or vection drum in our laboratory [35-37] and others.

**Figure 3:**
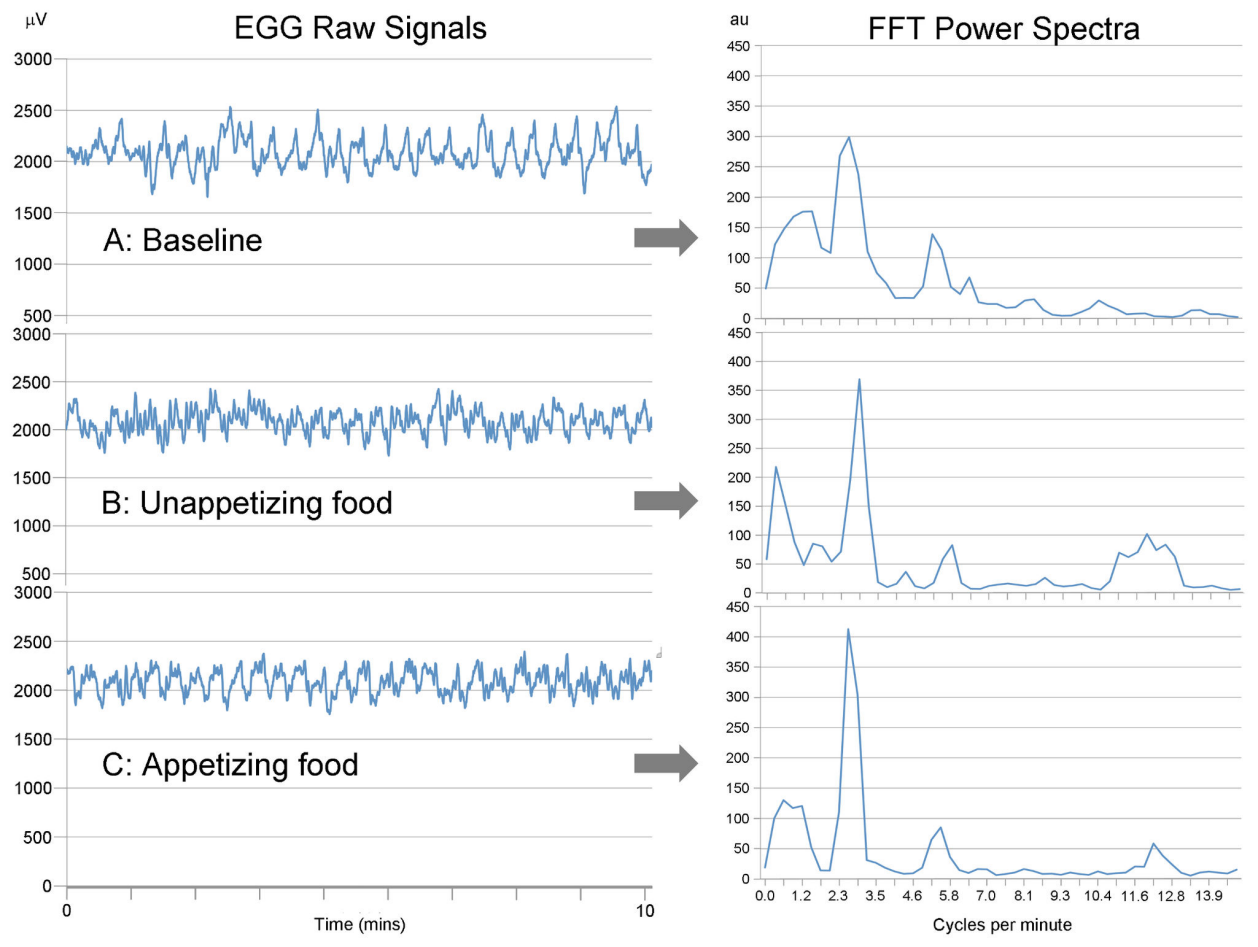
Sample raw EGG signals (myoelectrical activity, µV) (left) and respective FFT power spectra (arbitrary units) (right) from one volunteer (No. 28) during baseline recordings (A) and following imagining of unappetizing (B) and appetizing food (C) under hypnosis.

Recordings were screened visually for artifacts. Criteria for artifacts included: signals with improbable amplitudes (+/- 1000 μV) for myoelectrical activity of the stomach; and fast and sudden onset of signal change that did not fit to the surrounding signals. Segments containing artifacts were excluded from analysis. A continuous artifact-free EGG record with a minimum duration of 5 minutes to the maximum of the total experimental period was selected for analysis. Sample EGG signals and FFT power spectrum are given in Figure 3.

Segments of at least 5 min in length from baseline recordings, during imagination of one’s favorite and most disliked food, and following the drink-to-full test were analyzed with the FFT. In cases where a minimum artifact-free 5 minute period was not available, the participant was excluded from further analysis.

Of all recorded data segments from all 61 volunteers, those of 54 participants were acceptable in quality for EGG analysis, the remaining 7 (all from the hypnosis group) were completely excluded due to too many artifacts in the signal in all sections (see Figure 1).

### Statistics

Baseline data were compared between the groups using a t-test and chi^2^-test where appropriate. Well-being, hunger, and disgust ratings were compared across conditions (baseline, appetizing meal, unappetizing meal) by repeated-measures ANOVAs with the between-factor group (hypnosis, control). Correlations between measures were performed using Pearson’s R. EGG data were analyzed separately for each power band (brady-, normo-, and tachygastria) as well as for the ratio measure, by repeated-measures ANOVAs with the within-factor "time" (baseline, appetizing, unappetizing) and the between-factor "group" (hypnosis, control). Condition order was also examined as a covariate to identify any possible order effects (appetizing first, unappetizing first). Post-hoc t-tests were performed only as follow-up to significant ANOVA results, and therefore were not corrected to account for multiple comparisons.

All data are presented as mean ± SD or SEM, as indicated. The alpha level was set at 0.05 for all tests. Statistics were performed with SPSS version 19 (SPSS Inc., Chicago, IL, USA).

## Results

### Basic sample characteristics

Despite unequal (2:1) randomization to the hypnosis and control intervention, anthropometric, demographic, and psychometric characteristics were similar in both groups (Table 1). No comparisons between conditions for these characteristics reached statistical significance. 

**Table 1:** Basic characteristics of the study sample (mean ± SEM) (n.s.= not significant).

	Hypnosis	Control	Statistics
N	42	20	--
Age (years)	24.9 ± 0.6	25.5 ± 1.0	n.s.
Body Mass Index	21.7 ± 0.4	21.5 ± 0.7	n.s.
% smoking	12.2	5.0	n.s.
HGSHS*	6.29 ± 0.32	6.28 ± 0.39	n.s.
TAS-2**	55.9 ± 3.0	59.3 ± 4.7	n.s.
Well-being (baseline)	52.1 ± 0.7	52.0 ± 2.0	n.s.
Basic Brady (%)	36.7 ± 1.6	37.6 ± 1.9	n.s.
Basic 3cpm (%)	26.2 ± 1.0	26.8 ± 1.2	n.s.
Basic Tachy (%)	37.0 ± 1.5	35.4 ± 1.7	n.s.

*: Harvard Group Scale of Hypnotic Susceptibility (25); **: Tellegen Absorption Scale (26)

Twenty participants (50%) in the hypnosis group and 7 participants in the control group (35%) were identified as highly susceptible to hypnosis according to the normative values of the German version of the HGSHS [25] (Chi^2^ n.s.). 

### Well-being, hunger, satiety

**Figure 4:**
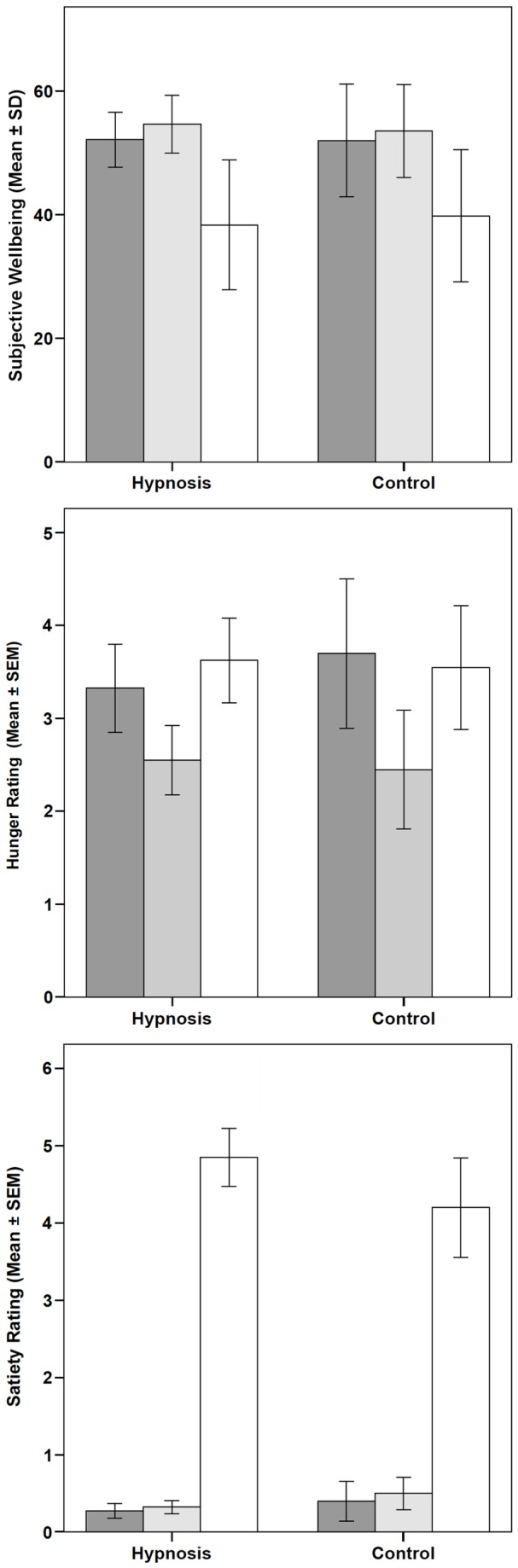
Subjective well-being scores (computed from 7 items rated on a visual analog scale) (A), and hunger (B) and satiety (C) ratings at three different time points (baseline and after both meal imaginations). Each block of columns represents data from hypnosis (left) and relaxation/concentration (right).

Imagination of appetizing and unappetizing food with and without hypnosis (control) significantly affected well-being (F=75.157, p<.001) and the ratings of hunger (F=5.743, p=.004) and satiety (F=139.7, p<.001) (Figure 4).

As can be in seen in Figure 4, the change from baseline was significant only for the unappetizing food, while baseline ratings and ratings under hypnosis or relaxation were not significantly different for the appetizing food. No group differences were found, and the sequence of imagined meals did not affect the outcome.

### Electrogastrogram

Under baseline conditions, distribution of the mean EGG power across the three frequency bands (bradygastria, normogastria, tachygastria) was 37.0 ± 9.2 %, 26.4 ± 5.9 %, and 36.6 ± 8.6 %, respectively, with no significant group differences (Table 1).

**Figure 5:**
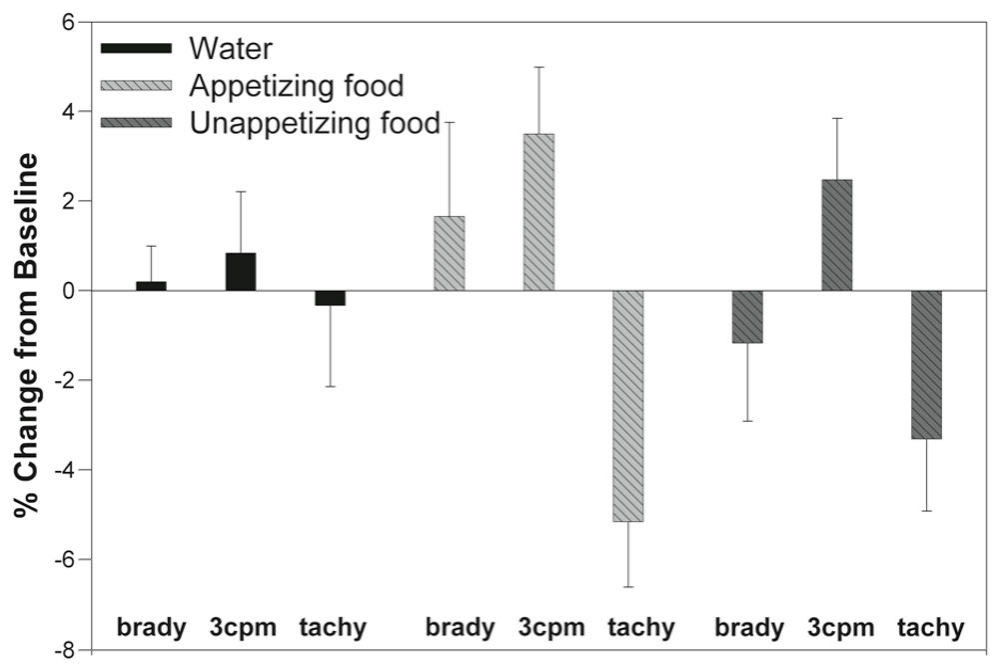
Change in EGG dominant power in three frequency bands (percent bradygastria, normogastria, tachygastria) from baseline during hypnosis. As can be seen, the response to a water load (left) is multiplied during imagining of an appetizing meal (middle) specifically in the tachygastria band, while it is less with the unappetizing meal (right). Note that error bars are understood to extend in both directions symmetrically.

**Figure 6:**
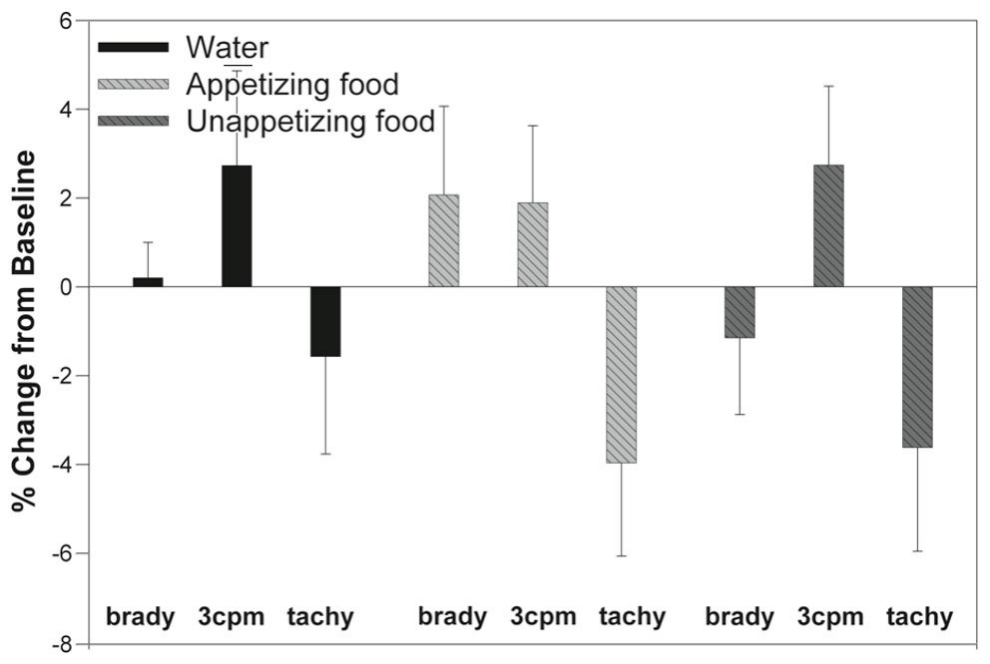
Change in EGG dominant power in all 3 frequency bands (percent bradygastria, normogastria, tachygastria) from baseline during relaxation. As can be seen, the response to a water load (left) is lower than with imagining of an appetizing meal (middle) and the unappetizing meal (right). Note that error bars are understood to extend in both directions symmetrically.

Ingestion of water "to full" resulted in a small decrease of tachygastria in favor of 3 cpm power but these effects did not reach statistically significant levels. In contrast, imagining of appetizing and unappetizing foods resulted in similar changes in the normo- and tachygastria bands that were highly significant (F=4.487, p=.034 and F=5.198, p=.004, respectively) (Figure 5, 6). The effect size of the decrease in tachygastria was stronger with the appetizing meal (eta^2^=.192) than with the unappetizing meal (eta^2^=.113). 

No main effects of group or sequence of meals could be seen, and the observed EGG effects were not different, when the group or sequence of meals was added as a factor to the ANOVAs (data not shown).

Similarly, the 3cpm-to-tachygastria ratio showed a significant shift towards higher values from baseline to the meal suggestions (F=6.652, p=.002) which was stronger for the appetizing than for the unappetizing meal, with no effect of hypnosis vs. control or of the meal sequence (Table 2).

**Table 2:** Ratio between the percentage of the normogastria and the tachygastria bands under different experimental conditions in the two groups.

	Hypnosis n=35	Control, n=19	Statistics
Baseline	0.800 ± 0.36	0.753 ± 0.27	n.s.
Appetizing	1.087 ± 0.62	1.085 ± 0.81	n.s.
Unappetizing Food	0.959 ± 0.44	1.013 ± 0.68	n.s.
Water Load Test	0.802 ± 0.28	1.014 ± 0.69	n.s.

### Inter-correlations and post-hoc analysis

The HGSHS and the TAS-2 did not correlate significantly (r=.16, p>.05), but TAS-2 scores correlated negatively with total well-being during unappetizing food imagination (r=-.37, p=.005). The HGSHS score positively correlated with the age of participants (r=.35, p=.01). 

A post-hoc analysis of hypnosis versus imagination effects on the EGG was performed for a subgroup of volunteers with HGSHS scores of 7 or higher, indicating high hypnotic susceptibility (n=20 in the hypnosis group and n=7 in the control group). It confirmed the results found (decrease in tachygastria and increase in the 3 cpm-to-tachy ratio, F=7.347, p=.002 and F=8.209, p=.009, respectively) for the entire group with no effect of group or sequence of meals (data not shown).

A further post-hoc subgroup analysis used a TAS-2 scale median split and analyzed participants with TAS-2 scores higher than 58 (18 in the hypnosis group and 11 in the control group). This resulted in weaker and non-significant responses of both hypnosis and imagination to imagination of appetizing and unappetizing food (data not shown).

## Discussion

Our study examined whether hypnosis induces gastrointestinal physiological effects, and if so, whether these effects are specific to hypnosis. Efficacy was examined by hypnotically suggesting eating an appetizing and unappetizing meal, and the specificity tested by using a concentration task as a control condition. While hypnosis was able to induce expected changes in the EGG (specifically a decrease in tachygastria) indicating high sensitivity, similar changes were seen with unspecific relaxation, indicating poor specificity of hypnosis. Susceptibility to hypnosis and "absorption" did not influence the outcome. 

This lack of a specific effect for hypnosis was somewhat surprising. However, it resembles a paradoxical finding in neuroscience: attempts to discriminate – on a central level - different modalities that induce analgesia to pain have found more similarities than differences in central activations and inactivations. This is despite the fact that there appear to be small but distinct differences, e.g. between acupuncture and placebo analgesia [38], and between the effects of hypnosis and other therapies of visceral pain [39], when investigated with functional magnetic resonance imaging. If, however, different psycho-physiological treatment modalities induce similar brain processes, it cannot be expected that peripheral physiological changes secondary to this central activation would be different. However, this post-hoc explanation is speculative, and would need independent empirical validation.

While the clinical efficacy of hypnosis in functional bowel disorders and related diseases has been supported [7-12], the underlying mechanisms of action remain obscure. Some studies have shown direct effects of gut-directed hypnosis on gastrointestinal (rectal) sensitivity [13,14,16,40], while others could not replicate these findings [15,32,33]. Some investigators have found changes in gastrointestinal motility [16,40,41] while others have not [13,15,42]. These previous works used clinical patients, and only a few included healthy controls. Furthermore, in most cases, motility and sensitivity were assessed prior to and after, either a single or a series of sessions of hypnosis, but not during hypnosis. Lack of sensitivity thus could be due to the fact that conditions other than the imagination of gut actions during hypnosis may contribute to intestinal sensory and motor alterations after hypnotherapy, e.g. altered cognitions and nutritional behaviors [43]. Furthermore, all except one study [41] used invasive measurement techniques that may have obscured potential effects and lowered sensitivity [44].

In contrast, we used healthy female volunteers in our study and recorded gastric myoelectrical activity of the stomach by a non-invasive technique, the EGG, during hypnosis. Studies that similarly assessed the influence of gut-specific and unspecific hypnosis on gastric functions in healthy volunteers, but prior to the era of hypnosis (e.g. 45), also found a general effect of hypnosis but no specific responses. However, these studies lacked valid measurement techniques to assess gut functions (gastric emptying and acid secretion) as well as appropriate control conditions.

To assess the potential specificity of findings, hypnosis-equivalent techniques may have to be used. The "cephalic phase" of food ingestion has been known to be potent to induce gastrointestinal physiological responses (saliva secretion) since Pavlov [46], and "sham feeding" techniques have been used in humans to induce gastric emptying [47], gastric acid [48] and pancreatic secretion [49,50], insulin [51] and Immunoglobulin A release [52]. Although Wolf and Wolf [53] previously described an increase in gastric motor activity when food was discussed with a fistulated patient, only limited attention has been given to cephalic influences on gastric motor activity.

Only a few studies have used the EGG during sham feeding. In 1989, Stern et al. [20] observed an increase in the amplitude of EGG signals after sham-feeding appetizing food. Except for being a shorter duration, the effect resembled that of eating real food. This effect was not noticed when participants experienced the procedure as disgusting. In a subsequent study, EGG reactions after sham-feeding appetizing food were compared to those after sham-feeding unappetizing food [54]. In contrast to the group sham-fed appetizing food, the 3 cpm power decreased in the group sham-fed unappetizing food. In the favorable food group the EGG amplitude at 3 cpm tended to increase more during the food-imagination period than in the baseline period. On the other hand, imagining unfavorable food resulted in a significant decrease in the EGG amplitude at 3cpm compared to baseline recordings. In a similar approach, Zhou et al. reported a decrease of normal 3cpm activity when viewing [21] or when imagining [22] unappetizing food. 

Whether "sham feeding" by chewing and spiting, e.g. two warm frankfurter sausages [55] can be considered "appetizing" at all, remains open for discussion. Since the landmark study by Schiller & Feldman [47], it has been known that purely imagining ones favorite food is superior in eliciting changes in gastric emptying as compared to viewing of food, smelling of food, or chewing of food. 

In consequence, the technique chosen in our study - imagining an appetizing and an unappetizing meal - should have been sufficient to elicit maximal gastric response under both conditions, during hypnosis and with relaxation/concentration. Our data confirm the findings by Zhou et al [22] and others [45,54], namely that imagining unappetizing food results in increased tachygastria and decreased normal 3 cpm activity, and these effects are less pronounced with appetizing food, irrespective of the condition under which imagery was induced (hypnosis or relaxation). Meissner et al. [55] has also found non-food effects of disgust, reporting that the percentage of bradygastria predicted disgust ratings in the case of highly arousing but non-food disgust pictures. When moderately arousing pictures were shown, disgust ratings were predicted by disgust sensitivity, which in turn was predicted by the percentage of bradygastria.

Several limitations of our study should be acknowledged. First, only healthy females were investigated and therefore we cannot assume that the findings would be similar in males or in patients with functional bowel disorders. The hypnosis protocol also differed in some aspects from those used in clinical and other experimental studies, and a different hypnotic protocol might produce different results [41]. The short-term hypnotic induction (10 minutes) and/or the use of audio-taped hypnotic instructions may also have influenced the results, despite contrary evidence, as discussed above (Method section). Finally, the clinical relevance of tachygastria is controversial [56], as is the EGG measurement during a gastric water load test [57]. Whether and why hypnosis is specifically effective in the treatment of functional bowel disorders still remains to be shown, and the quest for the mechanism of action is still open [58]. Whether hypnosis is effective in altering non-gastrointestinal autonomic functions [59] and other gastrointestinal functions and disorders [60-62] needs to be further examined in future research.
